# Intensity and Prevalence of Psychological Distress in Cancer Inpatients: Cross-Sectional Study Using New Case-Finding Criteria for the Hospital Anxiety and Depression Scale

**DOI:** 10.3389/fpsyg.2022.875410

**Published:** 2022-04-26

**Authors:** Barbara Muzzatti, Giulia Agostinelli, Francesca Bomben, Sara Busato, Cristiana Flaiban, Katiuscia Maria Gipponi, Giulia Mariutti, Sara Mella, Marika Piccinin, Maria Antonietta Annunziata

**Affiliations:** Unit of Oncological Psychology, Centro di Riferimento Oncologico di Aviano (CRO) IRCCS, Aviano, Italy

**Keywords:** anxiety, cancer inpatients, depression, hospital anxiety and depression scale, oncology, psychological distress

## Abstract

Psychological distress includes all negative subjective experiences elicited by a disease and its treatments. Since psychological distress in oncology is associated with negative outcomes, its detection and description are helpful for designing tailored supportive interventions. This study used the Hospital Anxiety and Depression Scale (HADS) to assess the intensity and prevalence of psychological distress (i.e., anxiety and depression) in cancer inpatients and examined the relationships between these variables and sociodemographic and clinical factors. An existing dataset of HADS results, from 2021 consecutive adult cancer inpatients at a single hospital, was analyzed. Only those questionnaires with complete responses were used. The intensity of anxiety and depression was determined from HADS sub-scores. The prevalence of anxiety and depression was calculated using, as case-finding criteria, cut-offs of ≥ 10 and ≥ 8, respectively. The mean HADS scores describing intensity were 7.3 for anxiety (*n* = 1,990) and 5.8 (*n* = 1,970) for depression. The prevalence rates for anxiety and depression were 26.6 and 28.6%, respectively. Among the 1,916 patients who completed both subscales, 17.2% had both anxiety and depression, 21.0% had either anxiety or depression, and 61.7% had neither. Gender, age, occupational status, and cancer diagnosis were associated with anxiety intensity or prevalence, while age, occupational status, and cancer diagnosis were associated with depression intensity or prevalence. Anxiety intensity was affected by the interaction effect between gender and diagnosis. Our study showed anxiety and depression being distinct entities, with more intense anxiety overall. From a research perspective, it reaffirms the usefulness for assessing both intensity and prevalence concurrently to gain a more detailed description of anxiety and depression.

## Introduction

Psychological distress includes all negative subjective experiences elicited by a disease and its treatments (National Comprehensive Cancer Network NCCN, 2003). It is reactive, situational suffering, with extreme variability in intensity, and it may complicate the natural processes of reaction and adaptation to a traumatic event, such as a cancer diagnosis (NCCN, 2003; Muzzatti et al., 2016). Psychological distress in cancer patients is associated with maladaptive coping, reduced quality of life, psychosocial morbidity, more unmet needs, poor treatment adherence, abnormal illness behavior, longer hospital stays, longer rehabilitation phases, family dysfunction, and impaired social relationships (Ballenger et al., 2001; Bringman et al., 2008; Grassi and Riba, 2009; Annunziata et al., 2012; VanHoose et al., 2015). Therefore, its detection and description are priorities in research and clinical practice.

In general, psychological distress is reported as a unique index or as the presence of anxiety or depression states (Vodermaier et al., 2009; Luckett et al., 2010; Mitchell, 2010; Muzzatti and Annunziata, 2012). The Hospital Anxiety and Depression Scale (HADS, Zigmond and Snaith, 1983) is a tool frequently used for detecting psychological distress in cancer patients (Muzzatti and Annunziata, 2012; Annunziata et al., 2020). It has been recently reported that HADS can be used to accurately determine the prevalence of anxiety and depression in cancer patients (Annunziata et al., 2020). According to this study, a score ≥ 10 on the HADS-A subscale distinguishes anxious participants from those who are not, while a score ≥ 8 on the HADS-D subscale distinguishes depressed participants from those who are not.

The availability of these new case-finding criteria for identifying anxious or depressed patients allows the collection of robust, updated data on the prevalence of these conditions in oncology. Therefore, this study determined the intensity and prevalence of psychological distress, in its two main components of anxiety and depression, in a large, heterogeneous sample of cancer inpatients at a single hospital. Moreover, it investigated the impact of sociodemographic and clinical factors on both distress components, and the relationships between their intensity and prevalence.

## Materials and Methods

### Participants

This study used data collected for a previous study (Annunziata et al., 2020) of 2,121 consecutive adult cancer patients, hospitalized for treatment. Inclusion criteria for the study were: age equal to or more than 18 years; good understanding of the Italian language; absence of mental disorders that could interfere with the psychological distress related to cancer; and absence of physical or sensory disabilities that would interfere with completing questionnaires.

This and the previous study were conducted in accordance with the Declaration of Helsinki, and the protocol was approved by the Ethics Committee of the IRCCS Centro di Riferimento Oncologico di Aviano (CRO), Italy. All participants gave written informed consent to participate in the study.

### Materials and Procedures

HADS (Zigmond and Snaith, 1983) was used to capture psychological distress in its two main components of anxiety and depression. It consists of two subscales: HADS-A for anxiety, and HADS-D for depression. Each subscale comprises seven items with a four-point ordinal response format. Scores range from 0 to 21 in each subscale, with higher scores indicating a higher intensity of anxiety or depression. On HADS-A a score ≥ 10 identified anxious persons, while on HADS-D a score ≥ 8 identified depressed persons (Annunziata et al., 2020).

Participants’ sociodemographic and clinical data were collected by a psychologist from clinical files. These data included age, gender, education level [compulsory (8 years), secondary, post-secondary], occupational status (employed vs. unemployed, homemaker or student), partnered status (married or cohabiting vs. non-partnered), and cancer diagnosis.

### Statistical Analyses

Only HADS subscales without missing data were analyzed. Anxiety and depression intensity was calculated, respectively, by totaling the HADS-A and HADS-D items. Differences in intensity between subgroups of patients by gender were tested for significance with Student’s *t-*test, while differences between subgroups according to other variables were tested with one-way univariate analysis of variance (ANOVA) followed by Bonferroni’s *post-hoc* test. To study the effect of age, patients were categorized into subgroups by decade (with patients aged 18–39 grouped together).

Since gender, age, and diagnosis are known to influence both anxiety and depression (NCCN, 2003; Muzzatti et al., 2016), two series of three two-way ANOVA (one using anxiety as the dependent variable and the other using depression) were performed to assess the presence of interaction effects due to two of these between-subject factors. Since breast cancer is primarily a disease of women, breast cancer patients were excluded from these analyses when gender was used as a between-subject factor.

The three-way ANOVAs between gender, age, and cancer type on both anxiety and depression were not performed, because such an analysis means generating 70 subgroups and so requires a much larger study population.

The prevalence of both anxiety and depression in the sample was obtained using the new case-finding criteria for HADS provided by Annunziata et al. (2020). Differences in the proportions of participants with and without anxiety or depression by each considered socio-demographic/clinical characteristic were tested by two series of six distinct chi-square tests.

A possible correlation between anxiety and depression scores was tested using Pearson’s correlation coefficient.

In all analyses, *p* < 0.05 (two-tailed) was pre-set for statistical significance.

The Statistical Package for the Social Sciences (SPSS), Version 20 was used to perform the analyses.

## Results

Of the 2,121 hospitalized cancer patients enrolled in the study, 1916 (93.7%) completed both subscales and 2,044 (96.4%) completed at least one HADS subscale (Table 1). The patients ranged in age from 18 to 84 years old. Their median age was 55 years, the mean age was 54.7, and only 43 cases were aged 18–30 years. The patients were predominantly female (72.9%), reflecting a high percentage of breast cancer cases (32.2%).

**TABLE 1:** Clinical and sociodemographic characteristics of 2,044 cancer inpatients.

Characteristic	Value
Age, years, median (range)	55 (18–84)
**Gender, *n* (%)**	
Male	553 (27.1)
Female	1,491 (72.9)
**Cancer type, *n* (%)**	
Breast	659 (32.2)
Genito-urinary	500 (24.5)
Digestive tract	301 (14.7)
Hematologic	238 (11.6)
Thoracic cavity including respiratory system	92 (4.5)
Oro-pharyngeal	57 (2.8)
Other	197 (9.6)
**Education, *n* (%)**	
Compulsory^a^	860 (42.1)
Secondary	910 (44.5)
Post-secondary	274 (13.4)
**Occupational status, *n* (%)**	
Employed	1,109 (54.3)
Unemployed, homemaker or student	791 (38.7)
Missing data	144 (7.0)
**Partnered status, *n* (%)**	
Married or cohabiting	1,530 (74.9)
Non-partnered	513 (25.1)
Missing data	1 (0)

*^a^Eight years.*

### Intensity and Prevalence of Anxiety

HADS-A was completed by 1,990 participants (Table 2).

**TABLE 2:** Anxiety intensity scores and prevalence according to HADS-A, by sociodemographic and clinical characteristic.

Characteristic	Patients, *n*	Score^a^	*P*	Score ≥ 10^b^	*P*
All patients	1,990	7.3 (3.8)	−	529 (26.6)	−
**Gender**			<0.001^c^		<0.001^e^
Male	539	6.3 (3.5)		90 (16.7)	
Female	1,451	7.7 (3.8)		439 (30.3)	
**Age group, years**			0.005^d^		0.127^e^
18–39	185	6.3 (3.6)		34 (18.4)	
40–49	482	7.3 (3.6)		131 (27.2)	
50–59	595	7.5 (3.8)		161 (27.1)	
60–69	541	7.5 (3.9)		151 (27.9)	
70 +	187	7.2 (4.1)		52 (27.8)	
**Cancer type**	\		<0.001^d^		<0.001^e^
Breast	645	7.8 (3.8)		194 (30.1)	
Genito-urinary	487	7.9 (3.9)		161 (33.1)	
Digestive tract	295	6.7 (3.8)		62 (21.0)	
Hematologic	231	6.4 (3.6)		39 (16.9)	
Thoracic cavity including respiratory system	88	7.6 (4.2)		31 (35.2)	
Oro-pharyngeal	56	6.4 (2.9)		9 (16.1)	
Other	188	6.4 (3.4)		33 (17.6)	
**Education**			0.181^d^		0.470^e^
Compulsory	833	7.3 (3.9)		216 (25.9)	
Secondary	887	7.2 (3.7)		233 (26.3)	
Post-secondary	270	7.7 (3.5)		80 (29.6)	
**Occupational status**			0.058^c^		0.034^e^
Employed	1,081	7.2 (3.6)		270 (25.0)	
Unemployed, homemaker or student	769	7.5 (4.0)		226 (29.4)	
Missing data	140				
**Partnered status**			0.226^c^		0.709^e^
Married or cohabitating	1,492	7.4 (3.7)		400 (26.8)	
Non-partnered	497	7.1 (3.9)		129 (26.0)	
Missing data	1				

*^a^Mean (SD); ^b^n (%); ^c^Student’s t-test; ^d^ANOVA; ^e^chi-square test.*

#### Intensity

The mean anxiety score was 7.3 (*SD* = 3.8). Female patients had higher anxiety scores than male patients (*p* < 0.001); the youngest patients (ages 18–39) had lower scores than the 40–49, 50–59, and 60–69 age groups (*p* < 0.005); and patients with breast or genitourinary cancer had higher anxiety scores than those with a cancer of the digestive tract, a hematologic cancer or other cancer (*p* < 0.001). No differences in anxiety scores were found according to education level, occupational status or partnered status.

#### Interaction Effects

To test the possibility of effects on anxiety score due to interactions among gender, age and diagnosis, two-way ANOVA was used. When gender and diagnosis were used as the independent variables, a significant interaction effect on anxiety score was found [*f*(5, 1,345) = 2.975; *p* = 0.011]. Although *post hoc* analysis did not reach statistical significance, a qualitative inspection of the data shows different scores among gender for several cancer types (see adictional materials). In contrast, no significant effect was found when gender and age [*f*(4, 1,990) = 1.701; *p* = 0.147] were considered or when age and diagnosis [*f*(24, 1,990) = 1.472; *p* = 0.065] were the independent variables.

#### Prevalence

According to the cut-off score of ≥ 10, the overall prevalence of anxiety was 26.6%. Significant differences in the proportions of participants with and without anxiety were found by gender (*p* < 0.001), occupational status (*p* = 0.035) and cancer type (*p* < 0.001), but not with age, education level or partnered status.

### Intensity and Prevalence of Depression

HADS-D was completed by 1,970 participants (Table 3).

**TABLE 3:** Depression intensity scores and prevalence according to HADS-D, by sociodemographic and clinical characteristic.

Characteristic	Patients, *n*	Score^a^	*P*	Score ≥ 8^b^	*P*
All patients	1,970	5.8 (3.8)	−	563 (28.6)	−
**Gender**			0.309^c^		0.469^e^
Male	537	5.6 (3.6)		147 (27.4)	
Female	1,433	5.8 (3.8)		416 (29.0)	
**Age group, years**			<0.001^d^		<0.001^e^
18–39	178	4.7 (3.3)		32 (18.0)	
40–49	485	5.4 (3.5)		124 (25.6)	
50–59	585	5.8 (3.7)		163 (27.9)	
60–69	537	6.2 (4.0)		173 (32.2)	
70 +	185	6.6 (3.9)		71 (38.4)	
**Cancer type**			0.018^d^		0.073^e^
Breast	639	5.6 (3.7)		172 (26.9)	
Genito-urinary	484	6.1 (3.8)		146 (30.2)	
Digestive tract	286	6.2 (3.9)		96 (33.6)	
Hematologic	225	5.2 (3.3)		49 (21.8)	
Thoracic cavity including respiratory system	91	6.3 (3.8)		31 (34.1)	
Oro-pharyngeal	57	5.7 (3.7)		15 (26.3)	
Other	188	5.7 (4.1)		54 (28.7)	
**Education**			0.132^d^		0.305^e^
Compulsory	829	5.9 (3.8)		247 (29.8)	
Secondary	872	5.6 (3.8)		234 (26.8)	
Post-secondary	269	6.0 (3.7)		82 (30.5)	
**Occupational status**			<0.001^c^		<0.001^e^
Employed	1,067	5.5 (3.6)		272 (25.5)	
Unemployed, homemaker or student	765	6.2 (3.9)		255 (33.3)	
Missing data	138				
**Partnered status**			0.196^c^		0.568^e^
Married or cohabitating	1,476	5.9(3.8)		427 (28.9)	
Non-partnered	493	5.6 (3.7)		136 (27.6)	
Missing data	1				

*^a^Mean (SD); ^b^n (%); ^c^Student’s t-test; ^d^ANOVA; ^e^Chi-square test.*

#### Intensity

The mean depression score was 5.8 (*SD* = 3.8). Employed participants had lower depression scores than patients who were unemployed, homemakers or students (*p* < 0.001). The two youngest subgroups (i.e., participants aged 18–39 and 40–49 years) had lower depression scores than the two oldest ones (60–69 and 70–84 years) (*p* < 0.001); in addition, participants 18–39 years old had lower depression scores than those aged 50–59 years (*p* < 0.001). Finally, patients with genitourinary cancer had higher depression scores than patients with a hematologic cancer (*p* = 0.002). No differences in depression scores were found according to gender, education level, or partnered status.

#### Interaction Effects

No significant interaction effect on depression score was found by two-way ANOVA using as independent variables gender and age [*f*(4, 1,970) = 0.527; *p* = 0.716], gender and diagnosis [*f*(5, 1,331) = 0.276; *p* = 0.926], or age and diagnosis [*f*(24, 1,970) = 0.831; *p* = 0.630].

#### Prevalence

According to the cut-off score of ≥ 8, the overall prevalence of depression was 28.6%. Significant differences in the proportions of participants with and without depression were found by age (*p* < 0.001) and occupational status (*p* < 0.001), but not by gender, education level, partnered status, or cancer type.

### Association Between Anxiety and Depression

A total of 1,916 participants completed both HADS subscales.

Anxiety and depression scores correlated, with Pearson’s *r* = 0.654 (*p* < 0.001).

The prevalence rates of anxiety and depression were similar (26.8 vs. 28.7%; *p* = 0.090, chi-square test). Overall, 17.2% of the group had anxiety and depression concurrently, whereas 21.0% had either “pure” anxiety or depression and 61.7% had neither (data shown as adictional material).

## Discussion

This study of a large, heterogeneous population of cancer inpatients found low intensities of anxiety (mean, 7.3 out of 21 on HADS-A) and depression (mean, 5.8 out of 21 on HADS-D). The composition of the study sample (i.e., patients with mental disorders were excluded) might had play a role in these data. However, certain subgroups of patients had higher intensities, so the prevalence rates of an anxiety state and a depression state were 26.6 and 28.6%, respectively.

Intensity scores for anxiety and depression correlated in the whole group. Overall, 17.2% of patients had both anxiety and depression while 61.7% had neither. These results indicate that anxiety and depression are distinct states, albeit associated with each other.

The finding that anxiety and depression are distinct states is important, since there is currently no agreement on how to define psychological distress. In fact, some researchers define anxiety and depression as the two main components of psychological distress, while others consider psychological distress a unique dimension (Vodermaier et al., 2009; Luckett et al., 2010; Mitchell, 2010; Muzzatti and Annunziata, 2012). By recording the prevalence of each state separately and of their concurrence, this study supports the view that anxiety and depression are distinct (although correlated) dimensions of psychological distress. Furthermore, this study shows that anxiety and depression vary with several important sociodemographic and clinical variables.

In this study, about two inpatients every 10 (17.2%) had anxiety and depression simultaneously, and another two every 10 (21.0%) had either anxiety or depression. These prevalence rates were calculated using new validated thresholds for an oncological population. Previous studies of cancer patients in different settings used different tools to assess the prevalence of anxiety and depression. The prevalence of anxiety has been reported to range between 10 and 48% (Stark et al., 2002; Keller et al., 2004; Gil et al., 2006; Strong et al., 2007; Annunziata et al., 2012; Linden et al., 2012), while rates of depression between 9 and 21% have also been reported (Keller et al., 2004; Gil et al., 2006; Strong et al., 2007; Annunziata et al., 2012; Linden et al., 2012).

Gender and cancer diagnosis affected both the intensity and prevalence of anxiety in this study, while age affected only intensity and occupational status affected prevalence. Regarding depression, age and occupational status affected both intensity and prevalence, while cancer type also affected intensity. Previous studies of the impact of sociodemographic and clinical factors on psychological distress in oncology are conflicting, due to differences in study populations (e.g., diagnosis, disease stage), methods and assessment tools applied, and the psychological distress definition itself (as a whole or in its main components). Our finding of gender differences in anxiety but not in depression only partially corroborates a previous study that reported gender differences in mental health (De Girolamo et al., 2006), and conflicts with previous studies that reported higher depressive states in females than males (Linden et al., 2012; Muzzatti et al., 2018). Younger participants in this study had less anxiety and depression than older patients, in accordance with one previous study (Sellick and Edwardson, 2007) but conflicting with other studies that found either higher depression and anxiety in younger patients (Linden et al., 2012; Naik et al., 2020; Smrke et al., 2020) or no age-related differences (Annunziata et al., 2012). For instance, the prevalence of moderate (26 vs. 20%) and severe (27 vs. 17%) distress was higher in 18–40-year-old cancer patients within 6 months of their diagnosis than in older patients (Smrke et al., 2020). Burgoyne et al. (2015) compared the distress levels of cancer patients aged 18–39, 40–64, and 65–90 years, and found that the youngest group had higher cancer-related distress than the oldest one but similar distress levels to the middle-aged group. Moreover, they found that gynecologic cancers were risk factors for high distress in the youngest patients; being divorced, single, or unemployed was risk factors for middle-aged group; and female gender was a risk factor for all age groups.

Several differences in the intensity and prevalence of both anxiety and depression were found in this study among groups based on cancer diagnosis. These are not new results. For instance, both depression and anxiety were more intense and wide-spread among patients with lung, hematological or gynecological cancer in two studies (Sellick and Edwardson, 2007; Linden et al., 2012). Another study found that women with genital cancers had a higher level and prevalence of distress than patients with other cancers (Mehnert et al., 2016). Finally, a recent database analysis found that gynecologic, respiratory, upper gastrointestinal, urinary, neuro-oncologic, and ear-neck-throat cancers were associated with higher distress than other cancers (Herschbach et al., 2020). Data on the relationship between cancer diagnosis and either anxiety or depression are useful for the identification of more vulnerable subgroups; however, differences in diagnostic categorization preclude comparisons across studies and, consequently, impair generalizations.

## Limitations and Strength

Even though this study enrolled over 2,000 patients, it was not possible to test the three-way interaction between gender, age, and cancer type, because such an analysis means generating 70 subgroups and so requires a much larger study population. We stratified the sample according to cancer types, other disease characteristics (e.g., stage, time since diagnosis, metastasis) should be considered in future research. Another limitation of this study is that confounding due to comorbidities or treatment side effects was not examined. We suggest that future studies take into consideration the effects of treatments, and the presence of symptoms such as pain and fatigue. Finally, the exclusion criteria of the study play a role on the generalizability of its findings. A future large study able to overcome all these limitations could provide valid norms for HADS according to the here used new case findings.

The main study strength consists of offering robust data on the intensity and prevalence of psychological distress in its two main components of anxiety and depression, calculated using new case-finding criteria for HADS. These data capture the scope of a phenomenon (the psychological distress), which is common, relevant and disturbing for all patients during the cancer trajectory. Consequently, the data provided here can serve as a starting point to plan prompt, tailored supportive interventions. From a research perspective, this study reaffirms the usefulness of assessing both intensity and prevalence concurrently, for a more detailed description of anxiety and depression.

## Data Availability Statement

The raw data supporting the conclusions of this article will be made available by the authors, without undue reservation.

## Ethics Statement

The studies involving human participants were reviewed and approved by CEUR—Friuli Venezia Giulia. The patients/participants provided their written informed consent to participate in this study.

## Author Contributions

MA and BM: conceptualization. GA, FB, SB, CF, KG, GM, SM, and MP: data curation. BM: formal analysis and writing—original draft. MA: writing—review and editing. All authors contributed to the article and approved the submitted version.

## Conflict of Interest

The authors declare that the research was conducted in the absence of any commercial or financial relationships that could be construed as a potential conflict of interest.

## Publisher’s Note

All claims expressed in this article are solely those of the authors and do not necessarily represent those of their affiliated organizations, or those of the publisher, the editors and the reviewers. Any product that may be evaluated in this article, or claim that may be made by its manufacturer, is not guaranteed or endorsed by the publisher.
