# Supplementary material for: Intensity and Prevalence of Psychological Distress in Cancer Inpatients: Cross-Sectional Study Using New Case-Finding Criteria for the Hospital Anxiety and Depression Scale
Source: Front Psychol. 2022 Apr 26;13:875410. doi: 10.3389/fpsyg.2022.875410 (PMC9087277; doi:10.3389/fpsyg.2022.875410)
Supplement: Supplementary file 1 [file Table_1.docx]

# *Intensity and prevalence of psychological distress in cancer inpatients: cross-sectional study using new case-finding criteria for the Hospital Anxiety and Depression Scale*

Barbara Muzzatti, Giulia Agostinelli, Francesca Bomben, Sara Busato, Cristiana Flaiban, Katiuscia Maria Gipponi, Giulia Mariutti, Sara Mella, Marika Piccinin, & Maria Antonietta Annunziata*

# Supplementary Tables

**Table 1**. Anxiety intensity scores according to gender and diagnosis

| **Diagnosis** | **Males** | | **Females** | |
| --- | --- | --- | --- | --- |
|  | **n** | **Mean (SD)** | **n** | **Mean (SD)** |
| Breast | 8 | 9.8 (5.0) | 637 | 7.8 (3.7) |
| Genitourinary | 32 | 5.4 (3.3) | 455 | 8.0 (3.8) |
| Digestive tract | 174 | 6.1 (3.5) | 121 | 7.5 (4.0) |
| Haematologic | 140 | 6.2 (3.5) | 91 | 6.7 (3.8) |
| Thoracic cavity including respiratory system | 52 | 6.6 (4.0) | 36 | 9.0 (4.2) |
| Oropharyngeal | 42 | 6.7 (2.9) | 14 | 5.5 (3.0) |
| Other | 91 | 6.1 (3.2) | 97 | 6.8 (3.6) |

**Table 2.** Distribution of anxiety and depression caseness, n (%)

|  | **No anxiety** | **Anxiety** | **Total** |
| --- | --- | --- | --- |
| **No depression** | 1183 (61.7) | 184 (9.6) | 1367 (71.3) |
| **Depression** | 219 (11.4) | 330 (17.2) | 549 (28.7) |
| **Total** | 1402 (73.2) | 514 (26.8) | 1916 (100) |
